# Transformation of European Ash (*Fraxinus excelsior* L.) Callus as a Starting Point for Understanding the Molecular Basis of Ash Dieback

**DOI:** 10.3390/plants10112524

**Published:** 2021-11-20

**Authors:** Anna Hebda, Aleksandra Liszka, Piotr Zgłobicki, Katarzyna Nawrot-Chorabik, Jan J. Lyczakowski

**Affiliations:** 1 Department of Plant Biotechnology, Faculty of Biochemistry, Biophysics and Biotechnology, Jagiellonian University, Gronostajowa 7, 30-387 Krakow, Poland; ana.hebda@gmail.com (A.H.); aleksandra.liszka@doctoral.uj.edu.pl (A.L.); piotr.zglobicki@uj.edu.pl (P.Z.); 2 Department of Forest Ecosystems Protection, Faculty of Forestry, University of Agriculture in Krakow, 29-Listopada Ave. 46, 31-425 Krakow, Poland; rlnawrot@cyf-kr.edu.pl

**Keywords:** *Agrobacterium tumefaciens*, ash dieback, callus, *Fraxinus excelsior*, transformation

## Abstract

The population of European ash (*Fraxinus excelsior* L.) is currently facing the risk of collapse, mainly due to ash dieback, a disease caused by a pathogenic fungus, *Hymenoscyphus fraxineus*. To facilitate studies into the molecular basis of ash dieback and design breeding strategies for a generation of resistant trees, it is necessary to develop tools enabling the study of gene function in *F. excelsior*. Despite this, a method for the genetic engineering of *F. excelsior* is still missing. Here, we report the first successful genetic transformation of *F. excelsior* callus and a selection process enabling the formation of stable transgenic callus lines. The protocol relies on the use of *Agrobacterium tumefaciens* to transform callus tissue derived from embryos of *F. excelsior*. In our experiments, we used the β-glucuronidase (GUS) reporter system to demonstrate the transformation of callus cells and performed RT-PCR experiments to confirm the stable expression of the transgene. Since ash dieback threatens the long-term stability of many native *F. excelsior* populations, we hope that the transformation techniques described in this manuscript will facilitate rapid progress in uncovering the molecular basis of the disease and the validation of gene targets previously proposed to be linked to the resistance of trees to *H. fraxineus* pathogenicity.

## 1. Introduction

European ash (*Fraxinus excelsior* L.) is a woody hardwood species with a broad geographical distribution and high-quality timber. These properties make *F. excelsior* both ecologically and economically important [1]. The European population of *F. excelsior* is currently experiencing significant pressure from the pathogenic fungus *Hymenoscyphus fraxineus,* which causes the dieback of ash trees [2]. The progress in understanding ash dieback has been greatly enhanced by solving *H. fraxineus* [3] and *F. excelsior* genomes [4] and by large sequencing projects of healthy and diseased trees [5]. However, rapid advances in uncovering the molecular basis of the disease remain impeded, largely due to a lack of genetic engineering tools for *F. excelsior.*

Study of *F. excelsior* trees resistant to *H. fraxineus* pathogenesis is one of the potential approaches for uncovering the molecular basis of ash dieback. The mortality of *F. excelsior,* due to dieback, can reach as much as 85% in certain populations [6]. Interestingly, typically around 1% of ash trees within affected forests show no visible symptoms of the disease, despite neighbouring trees dying of the infection [7]. Most of the available experimental data suggest that this resistance of *F. excelsior* to *H. fraxineus* has a significant genetic component and heritability [8,9,10], but some reports indicate that environmental factors may also influence tree resistance to the pathogen [11]. Specific techniques, such as spectroscopy of bark extracts, are being developed to screen for resistant trees [12], but importantly, the exact molecular reasons for the resistance of ash individuals to *H. fraxineus* remain virtually unknown [7]. Co-cultures of tree calli and fungal pathogens can be used to study the mechanism of infection [13,14]. Moreover, callus genetic engineering can be used to introduce disease resistant traits to plant genomes [15]. As such, the development of callus transformation techniques greatly facilitates reverse genetic studies that could enable the attribution of the *H. fraxineus* resistance phenotype, observed in some *F. excelsior* individuals, to specific gene alleles. However, such studies are currently impossible, since no technique exists for the transformation of European ash callus.

To address this issue, we developed a method for the transformation of *F. excelsior* callus tissue. In order to establish a transformation protocol for *F. excelsior* callus, we employed *Agrobacterium tumefaciens* as a vector of genetic material and used a plasmid encoding the GUS reporter expressed under the control of the 35S promoter. This promoter is active in a range of plant species and, together with GUS, it was successfully used in the transformation of other members of the Fraxinus genus [16,17,18]. In addition to GUS, our plasmid encodes a kanamycin resistance marker, which enabled us to identify selection conditions, allowing for the generation of stable transgenic callus lines.

## 2. Results

**Figure 1:**
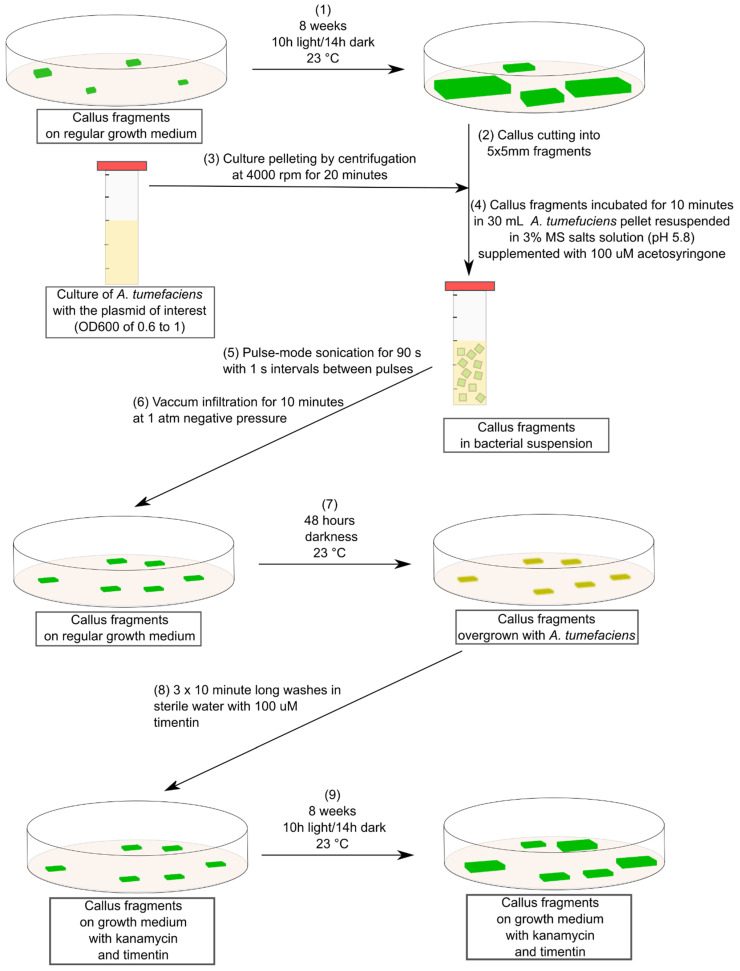
Schematic description of the transformation protocol for *F. excelsior* callus. AGL-1 *A. tumefaciens* was used for transformation of *F. excelsior* callus. A specific description of kanamycin and timentin concentrations used is provided in the manuscript text.

**Figure 2:**
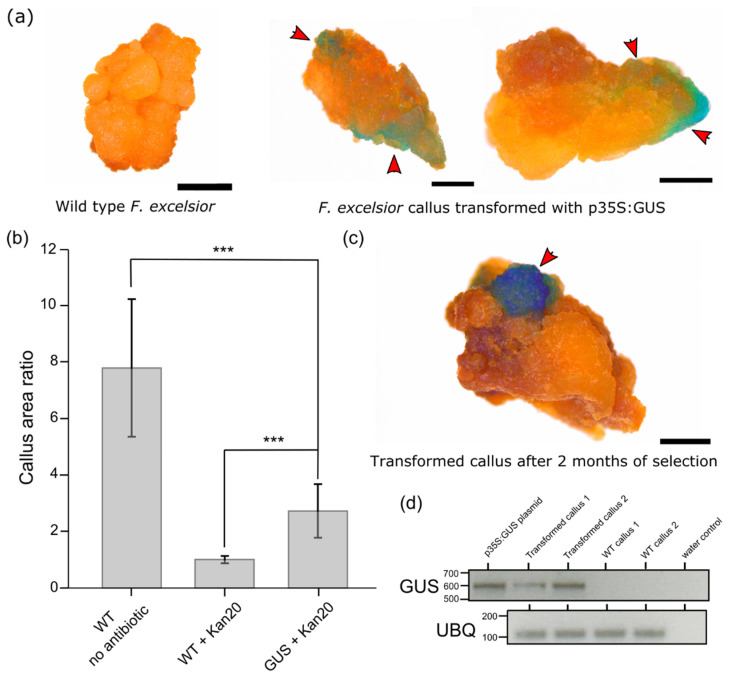
Transformation of *F. excelsior* callus. (**a**) Control (left) and transformed (right) callus fragments stained for GUS activity 10 days after incubation with AGL-1 *A. tumefaciens*. (**b**) Comparison of area increases for WT callus grown on medium without (WT no antibiotic) and with 20 µg/mL kanamycin (WT + Kan20) and for callus transformed with the pORE-R1:prom35S:GUS construct (GUS + Kan20). (**c**) Transformed callus fragment stained for GUS activity eight weeks after incubation with AGL-1 *A. tumefaciens*. (**d**) RT-PCR results for GUS (top) and UBIQUITIN (UBQ, bottom) amplified for two transformed and WT calli lines. For GUS, a purified pORE-R1:prom35S:GUS was used as a template in a positive control reaction. Water was used as a template in negative control reactions. Size bars in (**a**) and (**c**) correspond to 1 mm, red arrows indicate zones with GUS activity. The Student’s *t*-test was used to analyse the statistical significance in (**b**), *** denotes *p* ≤ 0.001.

Our transformation protocol (Figure 1) relies on the use of sonication and infiltration to deliver *A. tumefaciens* into the callus tissue. The presence of acetosyringone in the infiltration medium facilitates infection of callus cells by *A. tumefaciens* and the transfer of the plasmid-encoding reporter gene and the resistance marker. Our experiments employ the GUS β-glucuronidase reporter protein, which hydrolyses the glucuronic acid from the X-Gluc substrate, allowing for the formation of the insoluble, blue-coloured dimer molecule. As such, in our experiments, the appearance of blue colouration on the callus tissue is a result of GUS activity, which suggests the successful expression of the transgene. To assess if the proposed protocol is a viable approach for the transformation of *F. excelsior* callus, we assayed transformed and control calli for GUS activity ten days after performing sonication and infiltration procedures. Our analysis indicated that some of the callus fragments incubated with the p35S:GUS plasmid bearing *A. tumefaciens* developed a blue colouration (Figure 2a) and that none of the control callus fragments produced the pigment (Figure 2a). In our preliminary experiments, we tested a range of *A. tumefaciens* strains, including AGL-1, GV3101 and C58. Importantly, we were only able to observe blue colouration on the callus when AGL-1 *A. tumefaciens* was used to deliver the plasmid into the callus cells.

To enable the generation of stable genetically modified *F. excelsior* lines, it is necessary to develop a selection process for the transformed cells. In our experiments, we used the resistance to kanamycin to facilitate the selection process. Firstly, we analysed the growth of WT *F. excelsior* callus on a solid medium supplemented with a range of kanamycin concentrations (Figure S1). This experiment enabled us to establish that supplementation of the medium with 20 ug/mL kanamycin is sufficient to arrest WT callus growth (Figure S1). To quantify the efficiency of the selection process, we measured the area of change for WT calli grown on media with and without the addition of kanamycin (Figure 2b). Over an eight-week period, the WT callus increased its area by around eight times on the medium without kanamycin. Over the same period, callus fragments grown on the medium with 20 µg/mL kanamycin did not increase their area. We then measured the area increase for calli transformed with our construct, which, in addition to encoding the GUS protein, confers resistance to kanamycin. On average, the transformed calli increased their area more than two times over an eight-week period (Figure 2b). This indicates that cell division had occurred in the transgenic callus, which was likely enabled by the inserted transgene. To assay the activity of the transgene products eight weeks after transformation, we performed GUS staining on the callus fragments. We observed the blue colouration associated with GUS activity (Figure 2c and Figure S2) for parts of the transformed fragment, which may be attributable to the newly formed cell material.

Since it is possible that the GUS protein remains stable in cells only transiently transformed with the construct, we wanted to validate whether the GUS gene is expressed in callus grown on medium supplemented with kanamycin after an eight-week selection period. To this end, we performed RT-PCR for GUS on WT and transformed calli (Figure 2d). A GUS-specific amplicon was generated from the cDNA of transgenic callus and no amplification was observed for WT. As a housekeeping control, we amplified a 3′ end of *F. excelsior UBIQUITIN*. In this reaction, we observed an amplicon when using cDNA from both WT and the transgenic callus. As such, this experiment confirms that the GUS gene is still expressed in the transformed calli 8 weeks after co-incubation with *A. tumefaciens*.

## 3. Discussion

In this manuscript we describe a first protocol for the transformation of the callus tissue of *F. excelsior*. In our experiments, we used GUS as a reporter gene, expressed under the control of the 35S promoter, and a kanamycin resistance-selectable marker. Firstly, we evaluated the activity of the *GUS* transgene ten days after performing the agroinfiltration and sonication procedures on *F. excelsior* callus. We observed the blue-coloration associated with GUS activity only in calli incubated with *A. tumefaciens* harbouring the p35S:GUS plasmid. To assess if the transgene was stably integrated and maintained in the transformed cells, we developed an antibiotic-based selection process. By supplementing the growth medium with kanamycin, we were able to inhibit the growth of untransformed *F. excelsior* callus, while the calli fragments transformed with the plasmid did increase their sizes. By assaying the activity of the GUS enzyme eight weeks into the selection process, we demonstrated that the protein expressed from the transgene was still present in the transformed calli fragments. To confirm that the transgene is actively expressed eight weeks after transformation we performed RT-PCR analysis. This experiment indicated constant *GUS* mRNA production in the transgenic callus and provided further evidence for stable integration of the transgene into *F. excelsior* genome. Together, these experiments confirm that our protocol enables the transformation of the *F. excelsior* callus and the generation of stably transformed callus lines.

Previous publications have reported the transformation of other ash species, such as *Fraxinus pensylvanica* [16], *Fraxinus americana* [17] and *Fraxinus profunda* [18]. Our protocol shares many similarities with the previous experiments describing transformation of these other ash species. For example, like ours, previously published protocols apply both sonication and infiltration to facilitate *A. tumefaciens* transfer into plant cells. Moreover, in line with our results, for most investigated species, with a notable exception of *F. americana* [17], supplementation of the medium with 20 µg/mL kanamycin was sufficient to select the transformed cells. A key difference between our work and the previously published protocols lies in the type of plant material used for transformation; while other groups transformed hypocotyls, which were then used for the regeneration of transgenic callus, we transformed callus tissue directly.

In our experiments, it is important to distinguish transient and stable transformation events. Some of our transformed callus fragments produced blue pigment, associated with GUS activity, in multiple spots when stained ten days after infiltration with *A. tumefaciens*. Importantly, after eight weeks of incubation on the selection medium, a smaller number of zones with GUS activity was observed on calli (Figure 2a compared with Figure 2c). This suggests that, initially, multiple cells can be transformed in one callus fragment, but most of the initial transient GUS expression is likely to be lost over time. Similar results were observed for the hypocotyls of *F. pensylvanica* [16]. Importantly, stable maintenance of the transgene in only some of the transformed cells also might explain why the transgenic calli increased their area two times over the eight weeks of selection, while, over the same time, the WT callus grew more than eight times on the medium without kanamycin. Alternatively, this discrepancy in growth rate might be associated with the metabolic burden of transgene expression by the transformed cells.

At this stage it is important to consider how the transformation protocol for *F. excelsior* callus could be applied. One of the main interest points may lie in the validation of gene targets that have been linked to the resistance of *F. excelsior* to *H. fraxineus* pathogenesis. Previous genomic studies [5] showed that sixty-one out of the 192 strongest single nucleotide polymorphisms (SNPs) linked to low damage exerted by *H. fraxineus* are associated with *F. excelsior* genes for which homologues were demonstrated to be involved in plant responses to pathogens. Products of these genes include proteins likely involved in the control of mRNA translation efficiency or DNA repair. Mutations of these putative target genes or their overexpression could enable the identification of prime breeding targets for focusing the selection of resistant *F. excelsior* varieties. Such studies are now possible, using the transformation protocol presented in this manuscript.

In addition to enabling the study of specific genes that were previously linked to the resistance of *F. excelsior* to *H. fraxineus,* our method will facilitate the analysis of ash dieback at the level of whole processes. One such process that can be considered important in ash dieback is the interaction between *H. fraxineus* and the plant cell wall. Most plant pathogens need to penetrate cell walls as part of the infection process [19]. Indeed, the entry of *H. fraxineus* into ash leaf epidermal cells requires the mechanical penetration of primary cell walls [20] and polysaccharide cell-wall-degrading glycosyl hydrolases are the largest group of predicted secretory proteins of *H. fraxineus* [3]. As such, it is possible that the structure of its cell walls may contribute to the resistance of ash trees to *H. fraxineus*. Indeed, SNPs associated with ash resistance to dieback are located in the proximity of genes encoding a candidate boron transporter and a xyloglucan transglycosylase, both of which may be involved in primary cell-wall function [5]. Using the developed transformation protocol, it will be possible to test whether modifications of plant cell walls, which, for example, have been previously described to change the resistance of plants to pathogens [21], may alter the efficiency of *H. fraxineus* infection. The presence of such cell-wall variants in the natural populations of trees is unlikely; as such, our approach can be used for the identification of novel routes to generate resistant ash varieties. This will be further facilitated by the process of *F. excelsior* callus’ regeneration to seedlings that was recently described [22]. As recalcitrance to transformation is one of the main challenges in tree genetic engineering [23], the protocol described in this manuscript overcomes a major hurdle in understanding the molecular basis of ash dieback.

## 4. Materials and Methods

### 4.1. Plant Material Used and Culturing Conditions

Experiments were performed on in vitro-grown callus tissue. The full method of callus generation from zygotic embryos is described in a separate patent application [22]. Zygotic embryos, isolated from 300 mature *F. excelsior* seeds corresponding to individual *F. excelsior* genotypes, were used as initial explants. Calli derived from embryos were screened, and those showing good growth and maintaining high turgor were used for subsequent experiments. Allogamic seeds were obtained from the Kostrzyca Forest Gene Bank and originated from the Jarocin district of the General Directorate of State Forests located in western Poland (51°58′3.863″ N, 17°29′53.772″ E, 120 masl). Callus was grown on a medium described in a separate patent application; this medium is referred to as the “second solid medium” in the application, [22] before incubation with *A. tumefaciens* cultures. Calli of between 4 and 5 cm in diameter were cut into sections of up to 0.5 cm in diameter, which were used for the transformation. After co-incubation with *A. tumefaciens* calli were grown on the same medium as before the transformation. All calli used in the experiments were isolated from individual embryos and, as such, they represent different genotypes of *F. excelsior*. For growth experiments on the medium supplemented with kanamycin, 15 untransformed genotypes and 17 transformed genotypes were analysed. For the GUS experiments, calli from 10 individual genotypes were sectioned and subjected to the transformation protocol described. The transformation process was successfully replicated on three individual occasions.

### 4.2. Agrobacterium tumefaciens Culturing and Transformation

For the preparation of competent cells, a culture of AGL-1 *A. tumefaciens* was grown at 30 °C in LB supplemented with 100 µg/mL carbenicillin until OD_600_ reached ~0.6. Thereafter, the culture was chilled on ice, spun at 3000 RCF for 5 min at 4 °C and the pellet was re-suspended in 1 mL of 20-mM CaCl_2_. This suspension was aliquoted (100 μL/aliquot) and stored at −80 °C. For the *A. tumefaciens* transformation, an aliquot of chemically competent cells was thawed on ice. The liquid suspension was amended with 1 μg of plasmid DNA and incubated on ice for 10 min. Thereafter, the cells were frozen in liquid nitrogen and heat shocked at 37 °C for 5 min. This was followed by the addition of 400 μL of LB and outgrowth at 30 °C for 3 h. Following that, the cells were plated on LB agar amended with 100 µg/mL carbenicillin and 50 µg/mL kanamycin. Colonies of transformed *A. tumefaciens* were detected following 48 h of plate incubation at 30 °C. The presence of the plasmid-encoding GUS under the control of 35S promoter in the culture of *A. tumefaciens* used for transformation was confirmed with PCR (Figure S3) using primers for the GUS gene described in Table S1.

### 4.3. Callus Transformation and Selection

A detailed description of the transformation protocol is provided in Figure 1. In brief, callus fragments were placed in a culture of AGL-1 *A. tumefaciens* bearing the pORE-R1:prom35S:GUS plasmid [24] and sonicated (pulse mode sonication, with impulse applied at 1 s intervals for 90 s). Following sonification, bacteria were vacuum-infiltrated into the callus fragments. The infiltrated calli were placed on a solid medium and incubated in the dark for 48 h at 23 °C. After incubation, the callus fragments were washed in water supplemented with 100 µg/mL Timentin and placed on a solid medium amended with kanamycin (variable concentrations used) and 100 µg/mL Timentin. Callus growth was quantified with ImageJ.

### 4.4. GUS Staining and Imaging

A solution (100 mg/dm^3^) of X-Gluc (ThermoFisher Scientific) in 100-mM pH-7.0 sodium-phosphate buffer was used for GUS staining. The solution was vacuum-infiltrated into the callus fragments and the immersed calli were left overnight at 37 °C to allow for the GUS-catalysed reaction. Chlorophyll removal was performed with 96% ethanol. Callus images were obtained with a stereomicroscope (Leica S9i).

### 4.5. RT-PCR Analysis of Gene Expression

Total RNA was extracted from callus fragments grown on selective medium for eight weeks, using a commercial kit (Sigma, SpectrumTM Plant Total RNA). First-strand cDNA was synthesised using a polyA primers and RevertAid First Strand cDNA synthesis kit (ThermoFisher Scientific). For each RT-PCR reaction, 500 ng of cDNA was used. The primer sequences and PCR conditions used to amplify *GUS* and *UBIQUITIN* (*UBQ*) fragments are provided in Table S1. Sequences of the *UBIQUITIN* gene (Contig980) were identified using BLAST tool for the *F. excelsior* genome [4].

## Data Availability

All data in support of the findings is presented in the manuscript text and in the accompanying supporting information file.

## Supplementary Materials

The following are available online at https://www.mdpi.com/article/10.3390/plants10112524/s1, Figure S1—Selection conditions evaluated for *F. excelsior* callus. Figure S2—Further images of GUS activity in callus eight weeks after transformation. Figure S3—PCR analysis of *A. tumefaciens* cultures. Table S1—Primer sequences and RT-PCR conditions used.
