# Supplementary material for: Transformation of European Ash (Fraxinus excelsior L.) Callus as a Starting Point for Understanding the Molecular Basis of Ash Dieback
Source: Plants (Basel). 2021 Nov 20;10(11):2524. doi: 10.3390/plants10112524 (PMC8622397; doi:10.3390/plants10112524)
Supplement: Supplementary file 1 [file plants-10-02524-s001.zip › plants-1438997-supplementary.pdf]

Supporting Information file for:

Transformation of European ash (*Fraxinus excelsior* L.) callus as a starting point for understanding the molecular basis of ash dieback.

Anna Hebda[1], Aleksandra Liszka[1], Piotr Zgłobicki[1], Katarzyna Nawrot-Chorabik[2], Jan J. Lyczakowski[1,*]

[1] Department of Plant Biotechnology, Faculty of Biochemistry, Biophysics and Biotechnology, Jagiellonian University, Gronostajowa 7, 30-387 Krakow, Poland

[2] Department of Forest Ecosystems Protection, Faculty of Forestry, University of Agriculture in Krakow, 29-Listopada Ave. 46, 31-425 Krakow, Poland

[*] Corresponding author: Jan J. Lyczakowski (jan.lyczakowski@uj.edu.pl), ORCID: 0000-0002-7694-8629

Supporting information figure and table list and short legends:

Table S1 – Primer sequences and RT-PCR conditions used.

Figure S1 – Selection conditions evaluated for *F. excelsior* callus.

Figure S2 – Further images of GUS activity in callus eight weeks after transformation.

Figure S3 - PCR analysis of *A. tumefaciens* cultures

Table S1. Primer sequences used in this study. RT-PCR was performed with 10 uM forward and reverse primer concentrations using PCR-Mix Plus (A&A Biotechnology). For each reaction the initial melting was performed for 2 minutes at 95 °C. PCR was performed for 35 cycles with 30 seconds melting (95 °C), 45 seconds annealing (60 °C) and 90 seconds extension (72 °C). Final extension was performed for 5 minutes.

Primer name and target	Primer sequence (5'-3')
Fraxinus_excelsior_UBIQUITIN_For	GACCAGCAGCGATTGATCTTT
Fraxinus_excelsior_UBIQUITIN_Rev	GAGGACAAGATGGAGGGTAGAC
GUS_For	CAACGAACTGAACTGGCAGA
GUS_Rev	AGAGGTTAAAGCCGACAGCA

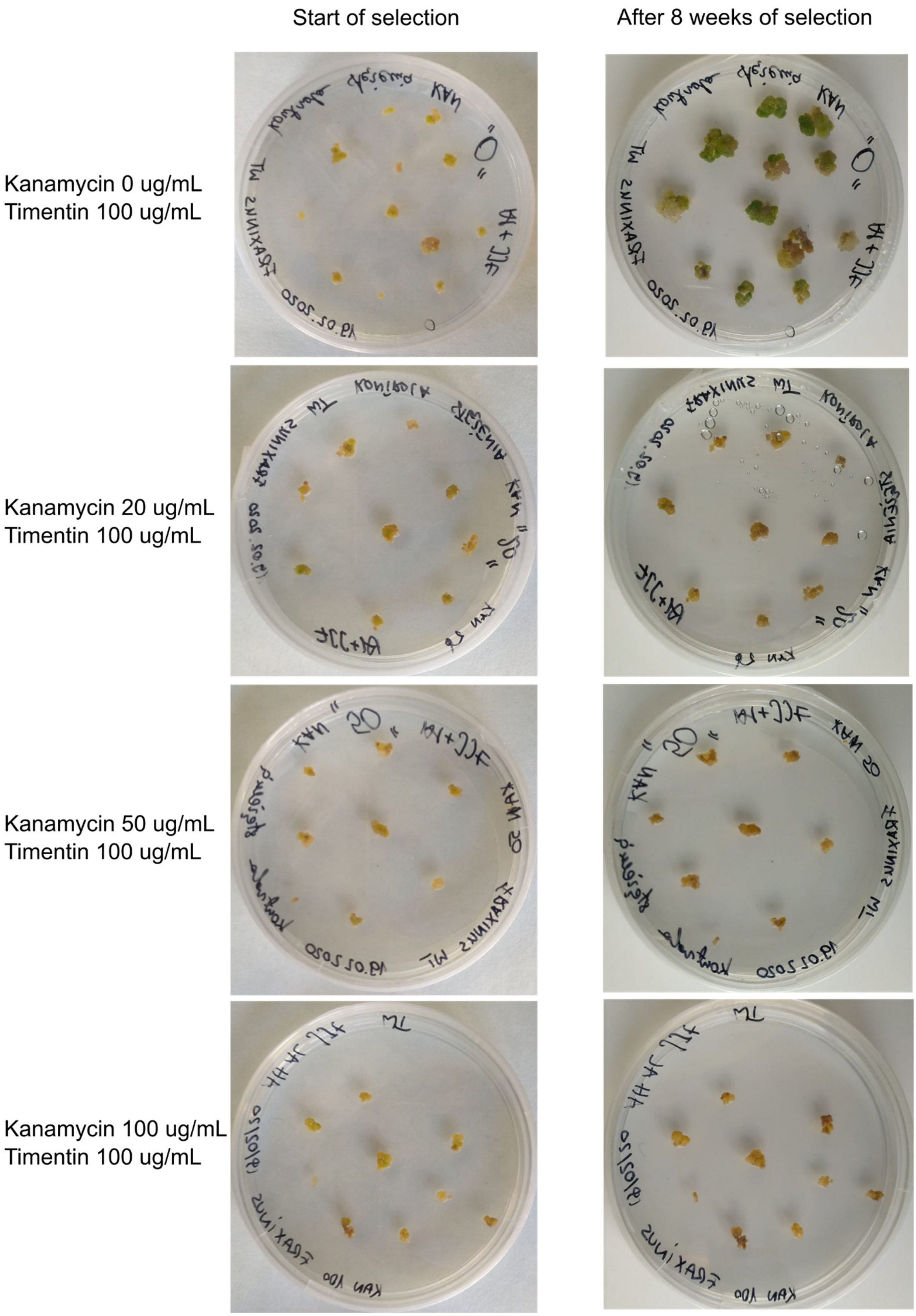

Figure S1. Selection conditions evaluated for *F. excelsior* callus. Kanamycin and timentin concentrations are provided for each image.

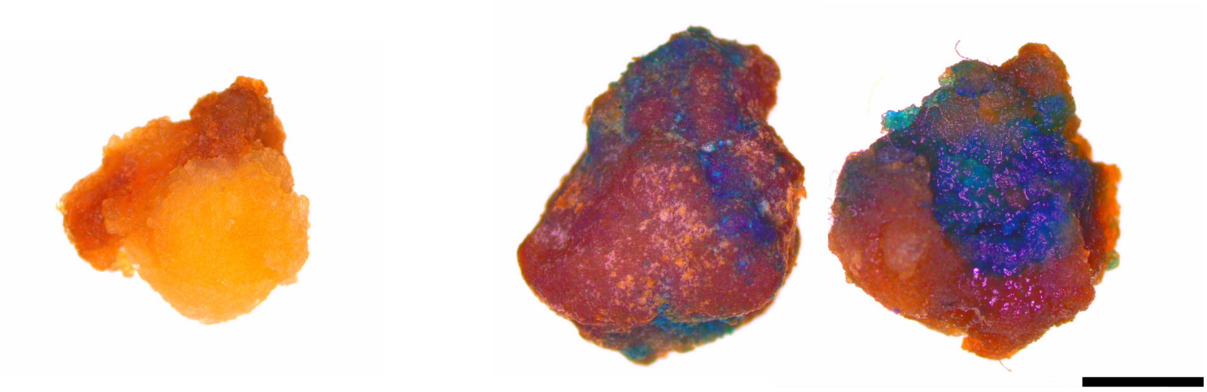

Wild type *F. excelsior* callus | p35S:GUS *F. excelsior* callus

Figure S2. Further images of GUS activity in callus eight weeks after transformation. Black size bar corresponds to 1 mm.

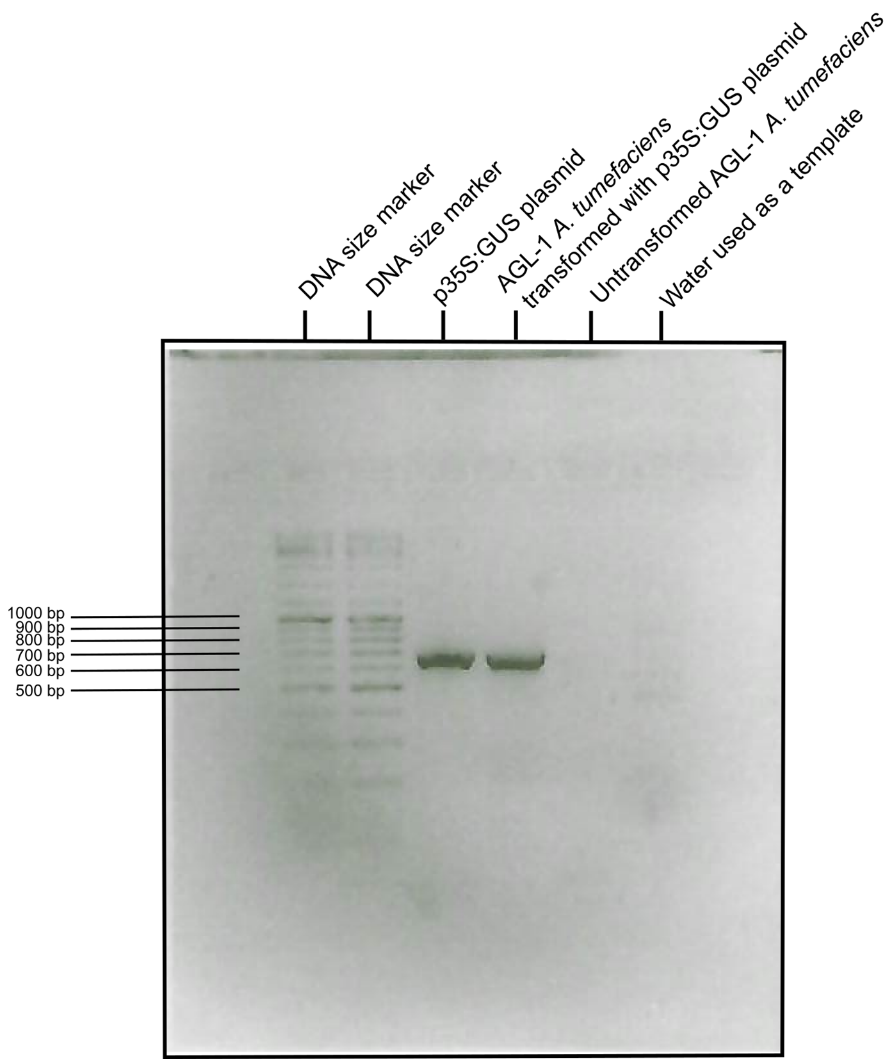

Figure S3. PCR analysis of *A. tumefaciens* cultures. Agarose gel electrophoresis of PCR products obtained with *GUS* specific primers (SI Table 1, same conditions used for amplification) using plasmid DNA (0.3 ng), transformed and untransformed AGL-1 *A.tumefaciens* cultures (10 µL of full-grown culture preheated to 98°C to release DNA, 1 µL of that used in PCR) or water as a template. Product of same size is observed when plasmid and transformed *A. tumefaciens* are used.
